# Nucleus incertus projections to rat medial septum and entorhinal cortex: rare collateralization and septal-gating of temporal lobe theta rhythm activity

**DOI:** 10.1007/s00429-023-02650-x

**Published:** 2023-05-12

**Authors:** Isis Gil-Miravet, Ángel Núñez-Molina, Mónica Navarro-Sánchez, Esther Castillo-Gómez, Francisco Ros-Bernal, Andrew L. Gundlach, Francisco E. Olucha-Bordonau

**Affiliations:** 1 Departamento de Medicina, Facultad de Ciencias de la Salud, Universitat Jaume I, CIBERSAM-ISCIII, S/N 12071 Castellón de la Plana, Spain; 2 Departamento de Anatomía, Histología y Neurociencia, Facultad de Medicina, Universidad Autónoma de Madrid, Madrid, Spain; 3 The Florey Institute of Neuroscience and Mental Health, Florey Department of Neuroscience and Mental Health and Department of Anatomy and Physiology, The University of Melbourne, Melbourne, Victoria Australia

**Keywords:** Arousal, Emotion, GABA, Hippocampus, Relaxin-3, RXFP3, Spatial memory

## Abstract

Nucleus incertus (NI) neurons in the pontine tegmentum give rise to ascending forebrain projections and express the neuropeptide relaxin-3 (RLN3) which acts via the relaxin-family peptide 3 receptor (RXFP3). Activity in the hippocampus and entorhinal cortex can be driven from the medial septum (MS), and the NI projects to all these centers, where a prominent pattern of activity is theta rhythm, which is related to spatial memory processing. Therefore, we examined the degree of collateralization of NI projections to the MS and the medial temporal lobe (MTL), comprising medial and lateral entorhinal cortex (MEnt, LEnt) and dentate gyrus (DG), and the ability of the MS to drive entorhinal theta in the adult rat. We injected fluorogold and cholera toxin-B into the MS septum and either MEnt, LEnt or DG, to determine the percentage of retrogradely labeled neurons in the NI projecting to both or single targets, and the relative proportion of these neurons that were RLN3-positive ( +). The projection to the MS was threefold stronger than that to the MTL. Moreover, a majority of NI neurons projected independently to either MS or the MTL. However, RLN3 + neurons collateralize significantly more than RLN3-negative (–) neurons. In in vivo studies, electrical stimulation of the NI induced theta activity in the MS and the entorhinal cortex, which was impaired by intraseptal infusion of an RXFP3 antagonist, R3(BΔ23-27)R/I5, particularly at ~ 20 min post-injection. These findings suggest that the MS plays an important relay function in the NI-induced generation of theta within the entorhinal cortex.

**Supplementary Information:**

The online version contains supplementary material available at 10.1007/s00429-023-02650-x.

## Introduction

The generation of cognitive maps in the entorhino-hippocampal pathway depends on associated electrophysiological features, particularly a prominent hippocampal theta rhythm, which is correlated with active exploration of a context. Theta activity is an oscillation of the local field potential of 4–12 Hz associated with the movement of an animal/person in the environment (Nuñez and Buño 2021). The medial septum (MS) is a major driver of hippocampal theta activity, and reversible inactivation of the MS in the rat results in a reduction in frequency and power of medial entorhinal (MEnt) theta as well as impairment of the hexagonal pattern of grid cells (Brandon et al. 2011; Koenig et al. 2011). This association between theta and tile fields in the MEnt has also been observed in non-human primates (Killian et al. 2012).

Brainstem afferents from the nucleus incertus (NI) to the MS and hippocampus may drive hippocampal theta activity, as electrical stimulation of the NI in urethane-anesthetized rats increased hippocampal theta and muscimol inactivation of the NI suppressed the hippocampal theta obtained by either sensory or electrical stimulation of the nucleus reticularis pontis oralis (Nuñez et al. 2006). In the same experimental set-up, intraseptal infusion of a relaxin-family peptide 3 receptor (RXFP3) agonist resulted in increased hippocampal theta activity. Relaxin-3 (RLN3) is a neuropeptide highly expressed in the rat NI and RXFP3 is present in the MS and hippocampus (Burazin et al. 2002; Tanaka et al. 2005; Ma et al. 2007). Furthermore, a preferential firing of NI neurons occurs during the ascending phase of the hippocampal theta cycle (Ma et al. 2013). There is also a synchronization of the hippocampus and the NI during theta-on states, and electrical stimulation of the NI evokes a reset of the phase of the hippocampal theta wave (Martínez-Bellver et al. 2017).

Theta rhythm has been proposed as a key element related to the plastic changes occurring during acquisition, consolidation and retrieval of memory, especially in entorhino-hippocampal circuits related to spatial navigation (Buzsáki and Moser 2013). In this context, manipulations of the NI and its signaling systems disrupt normal spatial memory processing. Transient inactivation of the rat NI impaired spatial memory in a Morris water maze (MWM) (Nategh et al. 2015). Depletion of RXFP3 in the mouse dentate gyrus resulted in disruption of spontaneous alternation in a T-maze paradigm, but did not affect MWM performance (Haidar et al. 2017). However, septal depletion of RXFP3 resulted in impairment of spatial navigation in the MWM (Haidar et al. 2019), indicating a possible differential role of septal vs hippocampal NI projections in spatial memory processing in the mouse.

Regarding the role of the NI in context perception associated with hippocampal theta rhythm, activation of GABA-ergic NI neurons disrupted the acquisition of context, while inactivation of these neurons enhanced retrieval of context conditioned memories in mice (Szőnyi et al. 2019). This action was, in part, mediated by a signal relay in the MS and via somatostatin-positive GABAergic interneurons in the hippocampal CA1 field. A reduction in hippocampal theta was also observed during NI activation (Szőnyi et al. 2019). These findings led to the proposal that the NI has a specific role in processing contextual features, inactivating irrelevant aspects and contributing to signal to noise ratio enhancement.

In a recent study, it was reported that activation of neuromedin-B-positive neurons in the mouse NI produced enhancement of theta rhythm activity and simultaneously increased locomotor activity; and that these actions were mediated by MS neurons (Lu et al. 2020). Notably, the vast majority of RLN3 neurons in the mouse NI also express neuromedin-B (Lu et al. 2020; Nasirova et al. 2020). Lastly, the entorhinal cortex is innervated by RLN3 + nerve fibers arising from the NI in rats (Tanaka et al. 2005; Ma et al. 2007; García-Díaz et al. 2021), mice (Smith et al. 2010) and non-human primates (Ma et al. 2009b).

Thus, the NI may drive or modulate entorhinal theta activity and this effect (or activity) could be mediated by the MS. In studies to explore this hypothesis, we simultaneously injected different retrograde tracers into the MS, dentate gyrus (DG) and entorhinal cortex and examined the level of their co-localization within the NI to study the relative contribution of each projection to global hippocampal function. In addition, in urethane-anesthetized rats, we studied whether electrical stimulation of the NI evoked theta activity in the MS and MEnt and whether RXFP3 antagonist administration into the MS altered the pattern of theta activity in the MEnt.

## Materials and methods

### Animals

This study used a total of 56 male and female Sprague–Dawley rats. The neuroanatomy studies are based on 36 Sprague–Dawley rats (Janvier, Le Genest-Saint-Isle, France) weighing 250–450 g. The electrophysiology studies employed 20 Sprague–Dawley rats (Iffa-Credo, Saint-Germain-Nuelles, France) weighing 300–400 g. All experimental procedures were approved by the Animal Welfare Ethics Committees of the Universidad Autónoma de Madrid (Spain) and Universitat Jaume I, Castellón (Spain) and were developed in accordance with the European Community Council Directive (86/609/EEC; 2010/63/EU), Spanish directive BOE 34/11370/2013, and local directive DOGV 26/2010.

### Tracing and surgical procedures

Rats were anesthetized with isoflurane (Isoflutek, 1000 mg/g, Karizoo, Barcelona, Spain) and placed in a stereotaxic apparatus (David Kopf Instruments, Tujunga, CA, USA) for surgery. Holes were drilled in the skull and cranial infusions of retrograde tracers were delivered into different brain regions using a Hamilton syringe (1 μl). The retrograde tracers used were FluoroGold (FG, 5-hydroxystabilamide; Cat No. 80014, Biotium, Hayward, CA, USA) and Cholera toxin-B (CTB, Cat No. 104, List Biological Laboratories Inc., Campbell, CA, USA). Injection coordinates, according to the atlas of Paxinos and Watson (2014), were (in mm) as follows: medial septum (MS) AP: + 0.6; ML: 0; DV: – 7; medial entorhinal cortex (MEnt) AP: – 6.8; ML: + 5; DV: – 8; lateral entorhinal cortex (LEnt) AP: – 6.8; ML: + 6.8; DV: – 7.6; and dentate gyrus (DG) AP: – 6.5; ML: + 6; DV: – 6.5. FG or CTB infusion volumes were 0.2 µl in all regions with an infusion rate of 0.1 µl/min and 5 min of diffusion time. After the infusion, the skin was sutured and all rats received analgesic treatment with meloxicam (Metacam, 2 mg/kg, 5 mg/ml, Boehringer Ingelheim, Barcelona, Spain). Rats remained undisturbed in their home cage for 7 days before perfusion.

### Brain fixation and sectioning

Rats were deeply anesthetized with sodium pentobarbital (Dolethal, 200 mg/kg, i.p.; Vetoquinol S.A., Madrid, Spain) and perfused transcardially with saline solution (0.9%; 250 ml) followed by fixative solution (4% paraformaldehyde in 0.1 M phosphate buffer (PB), pH 7.4) for 30 min (~ 500 ml). After perfusion, each brain was removed from the skull and immersed in the same fixative overnight at 4 °C. Thereafter, the brain was immersed in 30% sucrose in 0.01 M phosphate-buffered saline (PBS) pH 7.4 for 48 h at 4 °C. Coronal sections (40 μm) were cut using a freezing slide-microtome (Leica SM2000R, Leica Microsystems, Heidelberg, Germany). For each brain, 6 series of sections were collected and stored in cryoprotectant solution at – 20 °C.

### Immunofluorescence staining

For triple-labeling immunofluorescence, coronal sections were washed and incubated in blocking solution (2% normal donkey serum in 0.01 M PBS, 0.3% Triton X-100, pH 7.4) for 1 h and then incubated overnight at room temperature with 1:500 rabbit anti-FG (AB153-I, Merck Millipore), 1:5000 goat anti-CTB (Cat No. 227040, Calbiochem-Sigma-Aldrich, St Louis MO, USA) and 1:5 mouse anti-RLN3 (Kizawa et al. 2003; Tanaka et al. 2005; Ma et al. 2013) in blocking solution for 16 h. Tissue was then washed and incubated with 1:200 donkey anti-rabbit Alexa Fluor 488 (Jackson ImmunoResearch, West Grove, PA, USA), donkey anti-goat Cy3 (Jackson ImmunoResearch) and 1:200 donkey anti-mouse Alexa Fluor 647 (Jackson ImmunoResearch) for 2 h. Following further rinsing, sections were slide-mounted and coverslipped using Mowiol (Sigma-Aldrich).

### Image acquisition and analysis

Immunofluorescence images were captured with a confocal microscope (Leica DMi8, Leica Microsystems CMS GmbH, Wetzlar, Germany) with 20 × lenses and resolution of 1024 × 1024 dpi. Triple-labeling was captured with lasers 488 (laser intensity: 2.0%; gain: 675; offset: – 2), 561 (laser intensity: 2.0%; gain: 675; offset: – 3) and 633 (laser intensity: 2.0%; gain: 650; offset: – 2) that were constant for each analysis. Each image was formed by a stack of 20 images and was pre-processed applying a maximal projection process with Leica Application Suite LAS X (Leica Microsystems CMS GmbH). Infusion site images were compiled using multiple images collected with a 5 × objective using a tilescan option in the Leica Application Suite LAS X. Quantification of co-expression of FG, CTB and RLN3 in NI neurons (3 sections per rat) was conducted manually using the draw counter option of the Leica Application Suite LAS X.

### Electrophysiological recordings

Rats were anesthetized with urethane (1.4 g/kg, i.p.) and placed in a stereotaxic device (Kopf Instruments). Body temperature was maintained at 37 °C using a water-heated pad (Gaymar T/Pump, New York NY, USA). Supplementary doses of anesthetic were administered when a decrease was observed in the amplitude of the slow waves in the cortical field potential.

Trephine holes were drilled in the skull at preselected stereotaxic coordinates measured from bregma (Paxinos and Watson 2014). Field potentials were recorded through a macroelectrode (< 1MΩ, World Precision Instruments (WPI), Sarasota, FL, USA), targeting the MS (coordinates from bregma: 0.0 mm posterior and lateral, 7 mm ventral) and the MEnt (coordinates from bregma: – 8.6 mm posterior, 5 mm lateral and 7 mm ventral). Field potentials were filtered between 0.3 and 30 Hz, amplified and fed to a PC computer (1000 Hz sample frequency) for off-line analysis, using Spike 2 software (Cambridge Electronic Design, Cambridge, UK). The NI was electrically stimulated with a stimulation train of 0.2 ms single pulses at 50 Hz for 10 s, delivered by a Cibertec Stimulator (Madrid, Spain), using a concentric bipolar electrode (TM33CCINS-B; WPI). In some cases, to assess the role of the MS in relaying NI evoked entorhinal theta rhythm, inhibition of MS was obtained by injecting muscimol 0.1 µl of 1 mM muscimol (Sigma-Aldrich) dissolved in phosphate buffer (pH 7.4) through a 1-µl Hamilton syringe, and a stainless steel (30G) needle (Nuñez et al. 2006; Ma et al. 2009a).

At the completion of the experiment, each rat was trans-cardially perfused with saline followed by 4% paraformaldehyde. The brain was removed from the skull, cryoprotected in 30% sucrose in 0.01 M PBS and sectioned with a freezing slide-microtome. Sections were stained with Cresyl violet and coverslipped.

### Electrophysiology data analysis

Analyses of field potentials were performed off-line using Spike 2 software. The power spectrum of the MS or the Ent activity was calculated in 10 s intervals. The mean power density was calculated for two different frequency bands that constitute the more important oscillations in an anesthetized rat: delta (1–4 Hz) and theta (4–10 Hz) rhythms. Faster rhythms were neglected due to their low amplitude in anesthetized rats. The proportion of theta rhythm was calculated as the mean power in 10 s periods, before, during and after the NI stimulation train divided by the sum of delta and theta powers (theta/(delta + theta).

For comparison, the intensity of NI stimulation was set at twice the threshold to elicit theta rhythm in the hippocampal field potential for analysis (50–200 µA). Experiments in which higher intensities were needed to evoke a change in the MS field potential were rejected. The cross-correlation between MS and Ent field potentials was also calculated. Cross-correlation analyses provide quantitative information about the presence of theta rhythm in MS and Ent field potentials and more importantly, about the phase relationships between the rhythm in both structures.

## Results

### Neural tract-tracing experiments

**Fig. 1:**
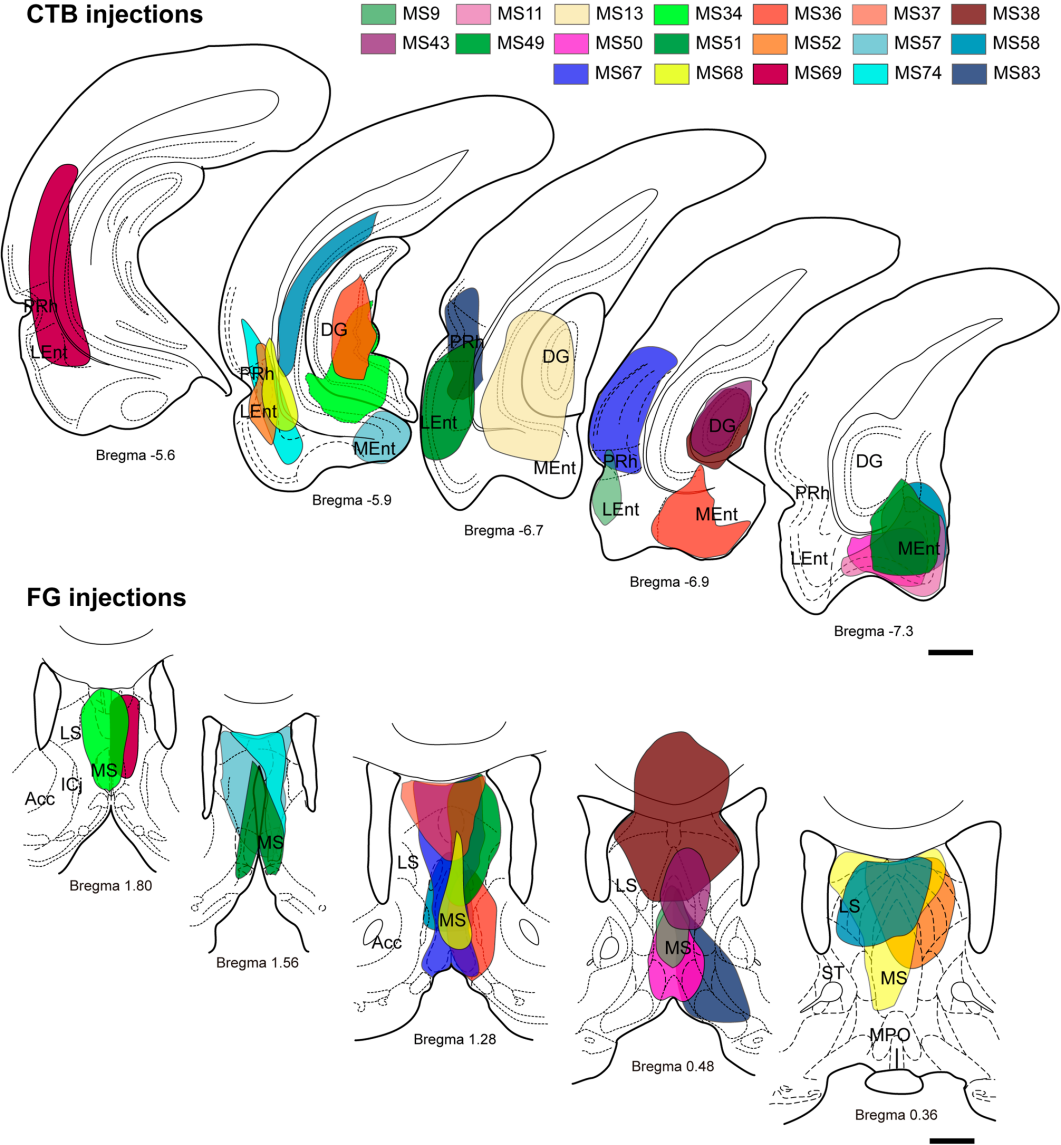
Anatomical distribution of tracers in cases analyzed in this study with combined injections of CTB in the MTL and FG in the MS. Some of the injections were administered into the same hemisphere of the brain, some into opposite hemispheres, and the remainder involved an FG injection into the midline, which affected both sides of the brain. Scale bars 5 mm

**Fig. 2:**
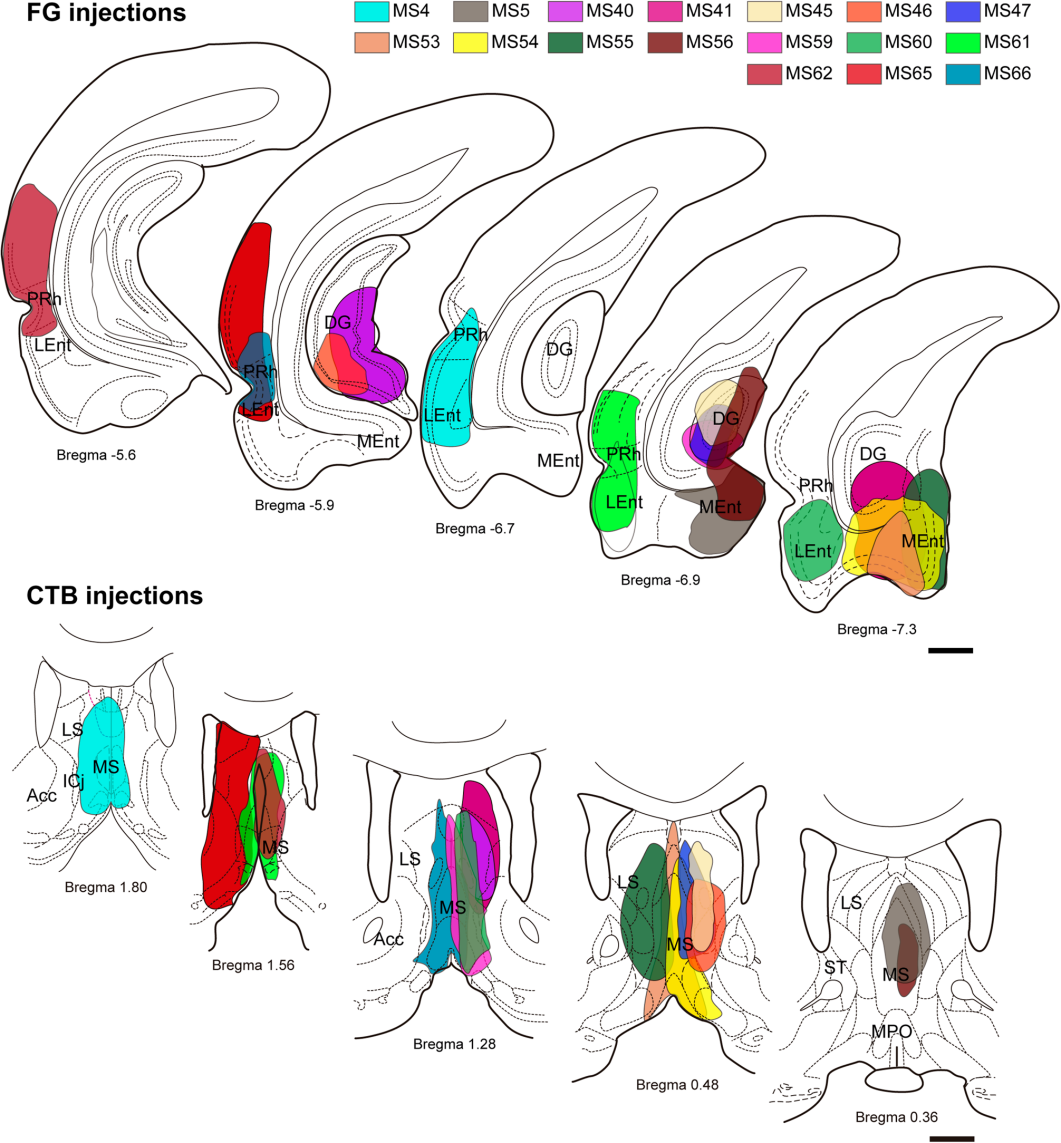
Anatomical distribution of tracers in cases analyzed in this study with combined injections of FG in the MTL and CTB in the MS. Some of the injections were administered into the same hemisphere of the brain, some into opposite hemispheres, and the remainder involved an FG injection into the midline, which affected both sides of the brain. Scale bar 5 mm

These studies were based on 36 cases in which the injections sites involved the combination of a FG injection into the MS and a CTB injection into the entorhinal/hippocampal areas, which were grouped under the term ‘medial temporal lobe’ (MTL; 19 cases; Fig. 1) or a CTB injection into the MS and a FG injection into the MTL (17 cases; Fig. 2 and Table 1). Therefore, there were three main neural tracer placement conditions as follows: (i) a tracer was injected into opposite sides of the brain; (ii) both tracers were injected into the same side of the brain; and (iii) the tracer injection into the MS was in the midline, and affected both sides of the brain. The number of rats (cases) that involved these three conditions were recorded (Table 1) and features of the organization of the projections observed are provided below.

**Table 1:** Cases analyzed in this study with different combinations of CTB and FG injections

Case	Sex	MS	LEnt	MEnt	DG	Laterality of tracer injection
MS60	M	CTB	FG	–	–	Opposite sides of the brain
MS62	F	CTB	FG	–	–	
MS65	F	CTB	FG	–	–	
MS4	F	CTB	FG	–	–	Midline of the MS
MS61	M	CTB	FG	–	–	
MS66	M	CTB	FG	–	–	
MS9	M	FG	CTB	–	–	
MS51	M	FG	CTB	–	–	
MS67	M	FG	CTB	–	–	
MS68	M	FG	CTB		–	
MS52	M	FG	CTB	–	–	
MS69	M	FG	CTB	–	–	
MS74	F	FG	CTB	–	–	Same side of the brain
MS83	F	FG	CTB	–	–	
MS5	F	CTB	–	FG	–	Opposite sides of the brain
MS41	M	CTB	–	FG	–	
MS50	F	FG	–	CTB	–	Same side of the brain
MS53	F	CTB	–	FG	–	
MS57	F	FG	–	CTB	–	
MS11	F	FG	–	CTB	–	Midline of the MS
MS13	F	FG	–	CTB	–	
MS36	F	FG	–	CTB	–	
MS49	F	FG	–	CTB	–	
MS54	F	CTB	–	FG	–	
MS55	M	CTB	–	FG	–	
MS38	M	FG	–	–	CTB	Opposite sides of the brain
MS40	M	CTB	–	–	FG	
MS43	M	FG	–	–	CTB	
MS56	M	CTB	–	–	FG	
MS58	M	FG	–	–	CTB	
MS59	M	CTB	–	–	FG	
MS34	F	FG	–	–	CTB	Same side of the brain
MS37	M	FG	–	–	CTB	
MS45	M	CTB	–	–	FG	Midline of the MS
MS46	M	CTB	–	–	FG	
MS47	F	CTB	–	–	FG	

Analysis of injection sites in the MS and a comparison of the diffusion areas of FG and CTB, revealed a mean (± SEM) diffusion area of 2.27 ± 0.30 mm^2^ and 2.17 ± 0.30 mm^2^ for FG injections and CTB injections, respectively. The distribution of FG and CTB diffusion areas passed the Shapiro–Wilk and Kolmogorov–Smirnov tests, and a comparison of the mean values revealed no difference between using CTB or FG as neural tract-tracers (*p* = 0.806; *t* = 0.2475, df = 36).

Assessment of the number of NI traced neurons per mm^2^ of tracer diffusion area revealed that FG injections in the MS resulted in 45.8 ± 11.3 labeled neurons in NI/mm^2^ of diffusion area, while CTB injections resulted in 52.7 ± 18.6 labeled neurons in NI/mm^2^ of diffusion area. Comparison of tracer densities revealed no significant differences between tracers (Mann–Whitney *U* test, *p* = 0.984).

#### Overall pattern of NI projections to the MS and MTL

**Fig. 3:**
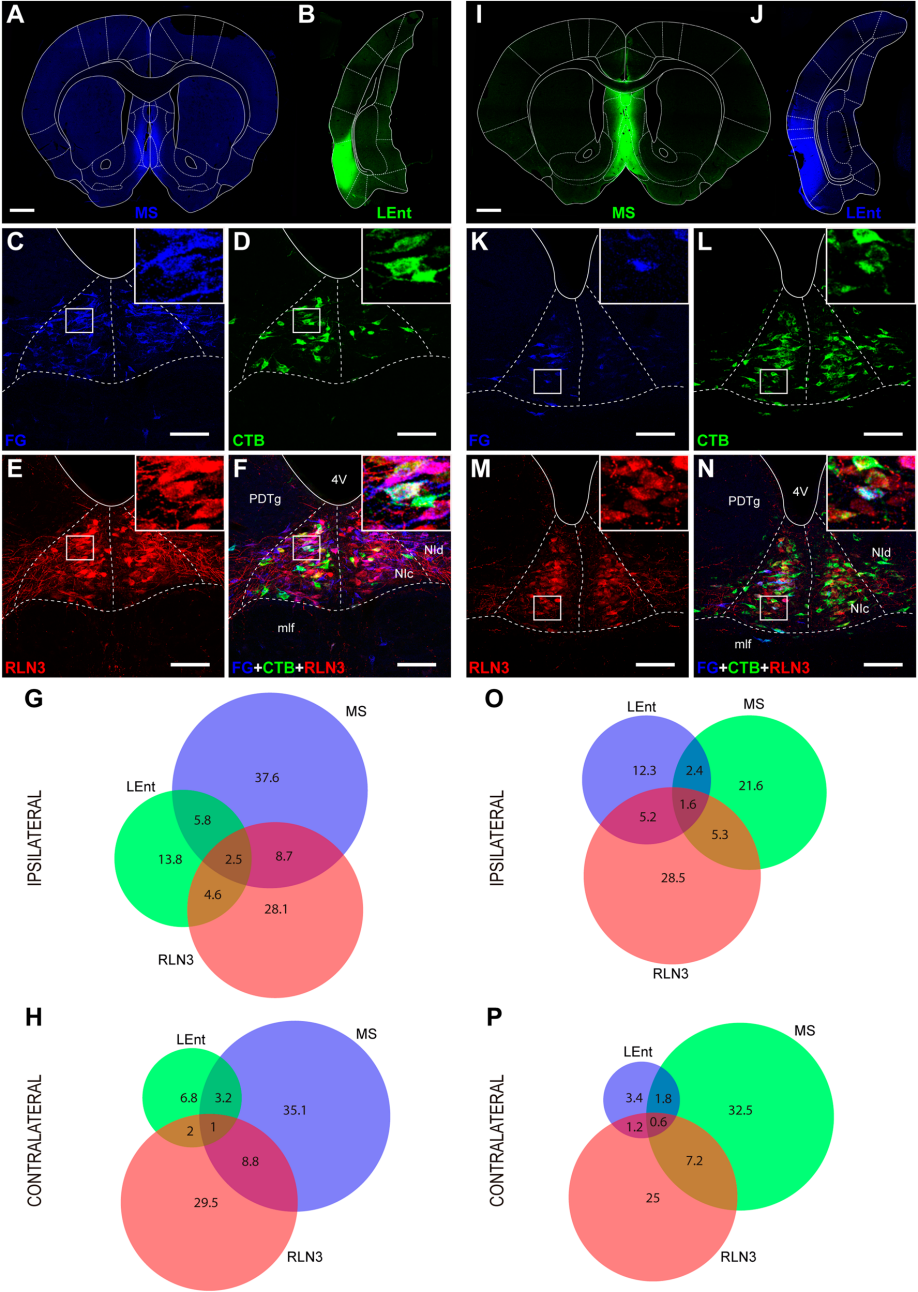
Representative images of the location of a FG injection in the MS (**A**) and a CTB injection in the LEnt (**B**). **C**–**F** Retrograde and RLN3 labeling in the NI resulting from the injections displayed in (**A**) and (**B**). **C** Retrograde FG labeling. **D** Retrograde CTB labeling. **E** RLN3 immunolabeling. **F** Multiple FG, CTB and RLN3 labeling. Scale bars, 1 mm (**A**, **B**, **I**, **J**). **G**, **H** Venn diagrams illustrating the proportional contributions of single-, double- or triple-labeled neurons to the projections from the NI to the MS and LEnt on the ipsilateral and contralateral sides. **I**, **J** Combinations of injections of CTB in the MS and FG in the LEnt. **K** Retrograde FG labeling. **L** Retrograde CTB labeling. **M** RLN3 labeling. **N** Multiple FG-CTB and RLN3 labeling. Scale bars, 100 µm (**C**–**F**, **K**–**N**). **O**, **P** Venn diagrams illustrating the proportional contributions of each type of neuron to the projections from the NI to the MS and LEnt on the ipsilateral and contralateral sides

In cases involving the combination of tracer injections into the MS and the LEnt, we observed retrograde-labeled neurons in the NI and the co-occurrence of tracer with RLN3 immunoreactivity (Fig. 3C–F). In cases involving tracer injection into the midline MS, retrograde labeling was detected on both sides of the NI in a similar proportion (~ 50%), while tracer injection into the LEnt produced mainly ipsilateral retrograde labeling (73%). A bilateral pattern of RLN3 immunoreactivity was consistent in all material examined (~ 50%). Irrespective of the combination of FG and CTB used, a larger proportion of the ascending projections was observed to target the MS than the LEnt (76% and 24%, respectively; Fig. 3G–H and O–P).

**Fig. 4:**
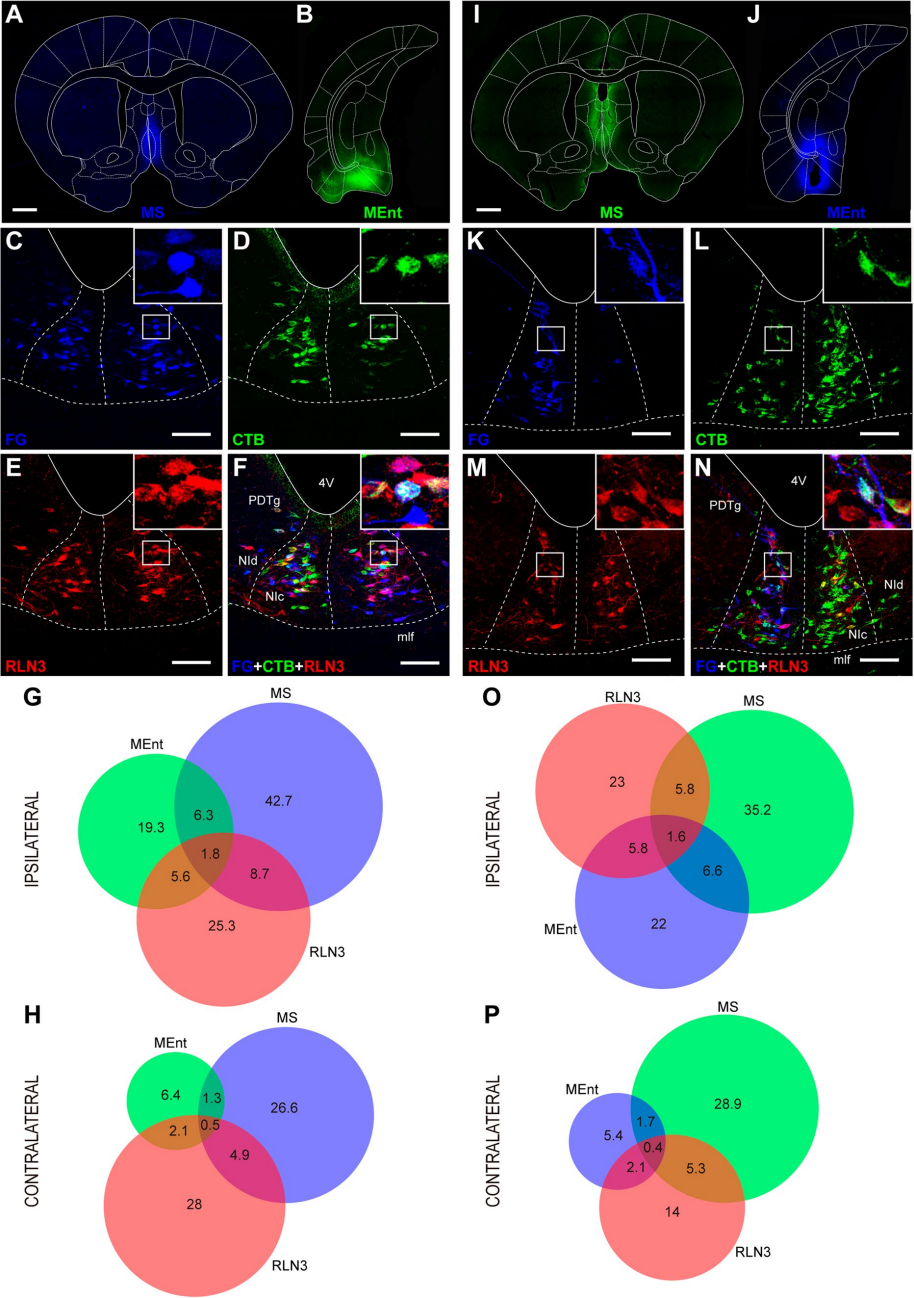
Representative images of the combination of injections into the MS and MEnt. **A**, **B** Injections of FG in the MS (**A**) and CTB in the MEnt (**B**). **C**–**F** Distribution of retrograde- and RLN3-labeling in the NI following the injections displayed in (**A**–**C**). Retrograde FG labeling in the two sides of NI. **D** Retrograde CTB labeling in the NI from an injection in the MEnt. **E** RLN3-positive neurons. **F** Overlay of multiple FG-CTB and RLN3 labeling. Scale bars, 1 mm (**A**, **B**, **I**, **J**). **G**, **H** Venn diagrams illustrating the proportional contributions of each subtype of neurons projecting to either MS, MEnt or RLN3. **I**, **J** Combinations of injections of CTB in the MS and FG in the MEnt. **K** Retrograde FG labeling. **L** Retrograde CTB labeling. **M** RLN3 labeling. **N** Multiple FG-CTB and RLN3 labeling. Scale bars, 100 µm (**C**–**F**, **K**–**N**). **O**, **P** Venn diagrams illustrating the proportional contributions of each subtype of neurons to the projections from the NI to the MS and MEnt in the ipsilateral and contralateral sides

In cases involving a combination of tracer injections into the MS and the MEnt (Fig. 4A, B and I, J), we observed a pattern of retrograde labeling in the NI (Fig. 4C, K) that was similar to that for the MS-LEnt (above). We observed labeled neurons in the NI and the co-occurrence of tracer with RLN3 immunoreactivity. In cases involving tracer injection into the midline MS, retrograde labeling was distributed on both sides of the NI, while tracer injections in the MEnt produced mainly ipsilateral retrograde labeling (77%; Fig. 4C–F and K–N). The bilateral pattern of RLN3 immunoreactivity was consistent (~ 50%; Fig. 4E, M). Regardless of the combination of FG and CTB used, a larger proportion of the ascending projection from the NI targeted the MS than MEnt on both the ipsilateral side (65% and 35%, respectively; Fig. 4G, O) and the contralateral side (81% and 19%, respectively; Fig. 4H and P).

**Fig. 5:**
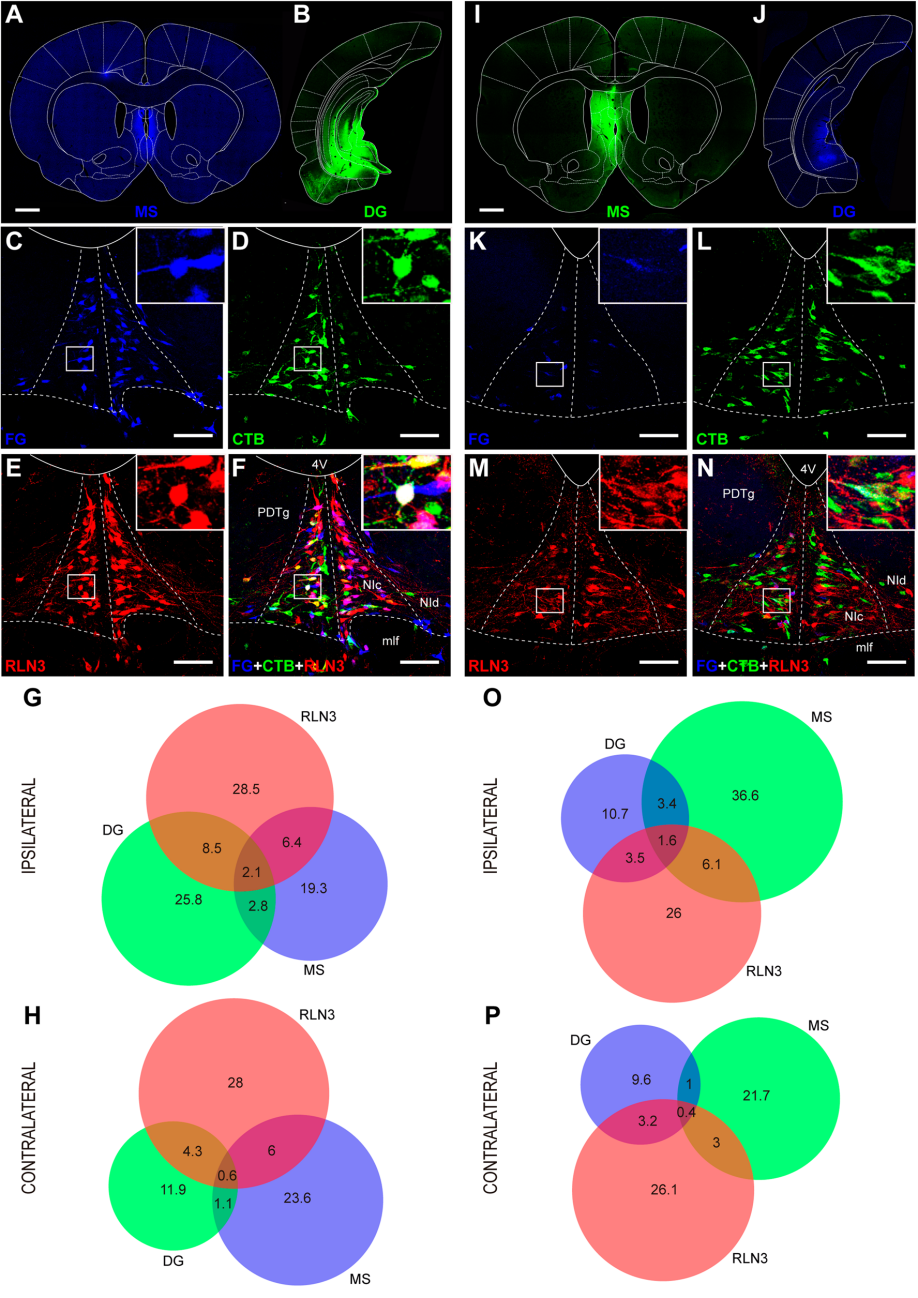
Representative images of the combination of injections into the MS and DG. **A** FG injection in the MS. **B** CTB injection in the caudal DG. **C**–**F** Distribution of FG, CTB and RLN3 following the injections displayed in (**A**–**C**). Retrograde FG labeling from the MS injection. **D** Retrograde CTB labeling from the DG injection. **E** RLN3-positive neurons in NI in the same section as (**C**, **D**, **F**). Multiple FG-CTB and RLN3 labeling. **G**, **H** Venn diagrams illustrating the proportional contributions of each subtype of neuron to the projections from the NI to the MS and DG in the ipsilateral (**G**) and contralateral (**H**) sides. Scale bars, 1 mm (**A**, **B** and **I**, **J**). Combinations of injections of CTB in the MS and FG in the MEnt. **K**. Retrograde FG labeling. **L**. Retrograde CTB labeling. **M** RLN3 labeling. **N** Multiple FG-CTB and RLN3 labeling. Scale bars, 100 µm (**C**–**F** and **K**–**N**). **O**, **P** Venn diagrams illustrating the proportional contributions of each subtype of neuron to the projections from the NI to the MS and MEnt in the ipsilateral and contralateral sides

In cases combining tracer injections into the MS (Fig. 5A, I) and the caudal DG (Fig. 5B, J), a similar pattern of labeling was observed to when injections were in the MEnt or LEnt. When a tracer injection was made into the midline MS, the retrograde labeling in the NI was quite evenly distributed on both sides (~ 50%; Fig. 5C, L), while tracer injection into the DG produced retrograde labeling concentrated in the ipsilateral side (72%; Fig. 5D, K). RLN3 immunolabeling was evenly distributed on both sides of the NI (~ 50%; Fig. 5E, M). Overlay images provided an account of single-, double- or triple-labeling (Fig. 5F, N). Analysis of the proportion of labeling indicated that the difference between the amount of retrograde NI labeling from the MS (54%) and the DG (46%) was less than the difference between the MEnt and LEnt (Fig. 5G–H and O–P). These findings indicate that the proportion of NI afferents to the DG is comparatively larger than to the MEnt or LEnt.

#### Patterns of collateralization of NI projections to MS or medial temporal lobe

**Fig. 6:**
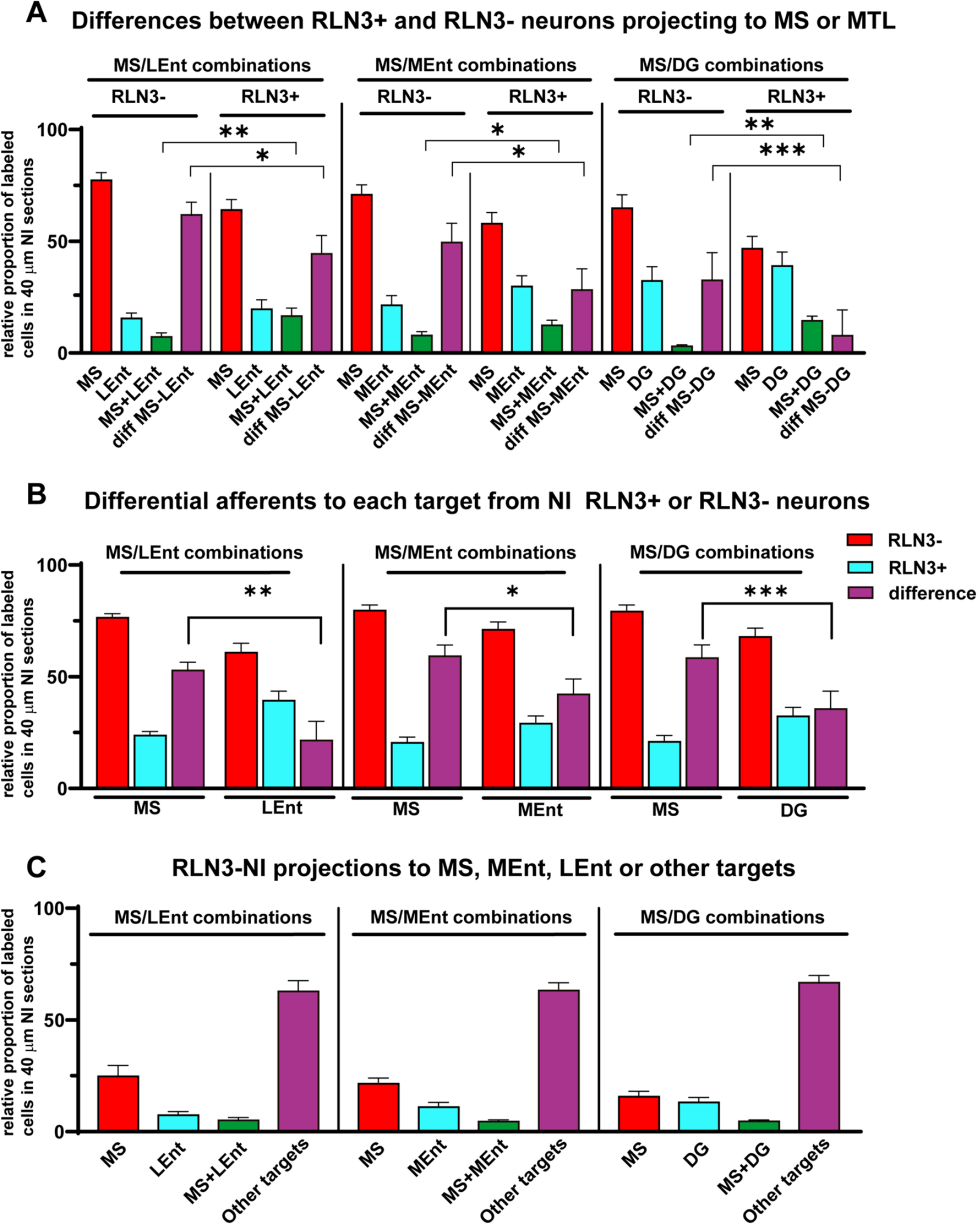
Comparative distribution of traced neurons positive or negative for RLN3-immunoreactivity. **A** Comparison between neurons projecting to the MS or either of the MTL areas. The third column corresponds to neurons projecting to both MS and MTL. The fourth column of each group is the difference between the labeling in the MS by MS retrograde tracing and the MTL retrograde tracing in the three group of injections MS-LEnt, MS-MEnt and MS-DG. **B** Occurrence of RLN3 in MS or MTL structures from the three groups of injections in the MS-LEnt, MS-MEnt and MS-DG; the third column in each group is the difference between RLN3-negative and RLN3-positive neurons. **C** Comparative proportions of RLN3-positive neurons projecting to any of the targeted structures or to other destinations in the three combinations of injections

A comparison of the relative proportion of neurons in the NI that project to either the MS or LEnt, MS or MEnt and MS or DG, revealed that NI neurons project more heavily to the MS (68.4%) than to the other MTL structures (31.6%; Fig. 6A, Table 2). The differences between the percentage of neurons projecting to the MS or the MTL followed a normal distribution.

**Table 2:** Relative percentages of retrogradely-traced neurons that projected to either MS or medial temporal lobe structures or collateralized and were or were not positive for RLN3 in the three combinations of MS/LEnt, MS/MEnt and MS/DG

RLN3–			RLN3 +		
MS/LEnt combinations					
MS	LEnt	MS-LEnt	MS	LEnt	MS-LEnt
77.3 ± 3.5	15.5 ± 2.4	7.2 ± 1.8	64.0 ± 4.7	19.6 ± 4.3	16.5 ± 3.7
MS/MEnt combinations					
MS	MEnt	MS-MEnt	MS	MEnt	MS-MEnt
70.8 ± 4.4	21.4 ± 4.3	7.8 ± 1.8	57.9 ± 4.9	29.7 ± 4.9	12.4 ± 2.3
MS/DG combinations					
MS	DG	MS-DG	MS	DG	MS-DG
64.8 ± 6.0	32.3 ± 6.3	3.0 ± 0.7	46.7 ± 5.5	38.9 ± 6.2	14.4 ± 2.1

Percentages are entered as mean ± standard error of the mean

For the combination of tracer injections in the MS and LEnt (Fig. 6A, first combination), the proportion of neurons projecting to the MS vs those projecting to the LEnt ranged from 5:1 in RLN3– to 3:1 in RLN3 + neurons. The percentage difference between neurons projecting to MS and to LEnt decreased from 61.8 ± 5.6 to 44.4 ± 8.2% (mean ± SEM) between RLN3– and RLN3 + neurons, and this change was significant (paired *t* test, *p* = 0.016; *t*_10_ = 2.841).

For the combination of tracer injections in the MS and MEnt (Fig. 6A, second combination), the proportion of neurons projecting to the MS vs those projecting to the MEnt ranged from 4:1 to 3:1 in RLN3– and RLN3 + neurons, respectively. The percentage differences between RLN3– and RLN3 + neurons projecting to the MS or MEnt decreased from 49.5 ± 8.5 to 28.2 ± 9.5% and this decrease was significant (*p* = 0.028; *t*_10_ = 2.566).

For the combination of tracer injections in the MS and DG (Fig. 6A, third combination), the proportion of cells projecting to the MS vs those projecting to the DG decreased from 2:1 for RLN3– neurons to 1:1 for RLN3 + neurons. The percentage differences between RLN3– and RLN3 + neurons projecting to MS or DG decreased from 32.6 ± 12.4 to 7.7 ± 11.6% and this decrease was significant (*p* < 0.001; *t*_10_ = 5.416).

These data reveal that RLN3 + neurons project more heavily to the medial temporal lobe than other (RLN3–) NI neurons.

In contrast, the degree of collateralization was low, ranging from 3 to 7% for RLN3– NI neurons projecting to both MS and medial temporal lobe and 12–16% for RLN3 + neurons projecting to MS and medial temporal lobe (Fig. 6A; Table 2). In the three combinations, the percentage of RLN3 + neurons that projected to MS and medial temporal lobe was double the corresponding RLN3– neurons. The differences were significant for all three combinations, i.e. the MS-LEnt combination (*p* < 0.01, *t*_14_ = 2.8), the MS-MEnt combination (*p* = 0.02, *t*_10_ = 2.32) and the MS-DG combination (*p* < 0.01, *t*_10_ = 6.29). Thus, RLN3 + neurons in the NI collateralize more than other NI neurons projecting to the MS and medial temporal lobe.

An analysis of the proportions of traced neurons that contained RLN3 immunoreactivity revealed that globally, 78.17 ± 1.44% (mean ± SEM) of NI neurons did not display RLN3 immunoreactivity, while 21.83 ± 1.44% contained RLN3 immunoreactivity, and these proportions were similar in the three experimental groups (i.e. combined injections in MS/LEnt, MS/MEnt and MS/DG; Suppl. Table 1). However, in each experiment, there were differences between MS projecting and medial temporal lobe projecting RLN3– neurons (single-labeled with tracer) and RLN3 + neurons (double-labeled with tracer and RLN3 in each tracer combination; Fig. 6B, Suppl. Table 1).

In the LEnt/MS tracer combination (Fig. 6B, first group), the difference between MS projecting RLN3– and RLN3 + neurons was larger than the difference between LEnt projecting RLN3– and RLN3 + neurons (*p* = 0.005; *t*_11_ = 3.524). Similarly, in the MEnt/MS tracer combinations (Fig. 6B, second group), the difference between MS projecting RLN3– and RLN3 + neurons was larger than the difference between LEnt projecting RLN3– and RLN3 + neurons (*p* = 0.016; *t*_10_ = 2.891). Finally, in the DG/MS combination, the difference between the proportion of RLN3– and RLN3 + neurons labeled by MS vs DG tracer injections was significant (*p* < 0.001; *t*_10_ = 5.015; Fig. 6B).

In conclusion, while the proportion of RLN3 + neurons in the NI that project to the MS was ~ 1:4, this proportion was reduced to ~ 1:3 for RLN3 + neurons projecting to the medial temporal lobe (either the LEnt, MEnt or DG).

We also analyzed the proportion of RLN3 + neurons projecting to any of the targeted structures or to other destinations (Fig. 6C). In the combination of MS-LEnt injections, RLN3 + neurons projecting to the MS represented 24.8 ± 4.8% of the total NI RLN3 + neurons. RLN3 + neurons projecting to LEnt were 7.4 ± 1.7%, and 5.1 ± 1.2% of projections to both MS and LEnt were RLN3 + . The remaining RLN3 + neurons (i.e. 62.8 ± 4.8%) in the NI did not project to either MS or LEnt. In the combination of MS-MEnt injections, the RLN3 + neurons that projected to the MS averaged 21.6 ± 2.6%, which was approximately double the percentage of RLN3 + neurons that projected to MEnt, i.e. 11.0 ± 2.1%. Neurons triple-labeled for both tracers and RLN3 were 4.4 ± 0.8%. Again, the percentage of RLN3 + neurons that did not project to either MS or MEnt was 63.0 ± 3.5%. Finally, in the combination of MS-DG injections, the proportion of RLN3 + neurons that projected to MS was 16.1 ± 2.6%, similar to the proportion of RLN3 + neurons that project to the MEnt. In this combination, triple-labeled neurons were 4.7 ± 0.7%. The percentage of RLN3 + neurons that did not project to either MS or DG was 63.9 ± 2.3%.

It follows from these results that ~ 40% of the RLN3 projections originating in the NI reach the septohippocampal axis and theta rhythm/spatial memory-associated centers. RLN3 + fibers arising from the NI also reach a variety of other centers, including the prefrontal cortex, amygdala, hypothalamus and brainstem, which may shape cognitive functions associated with either emotional or metabolic functions.

#### Distribution of retrograde labeling in the NI subnuclei

**Fig. 7:**
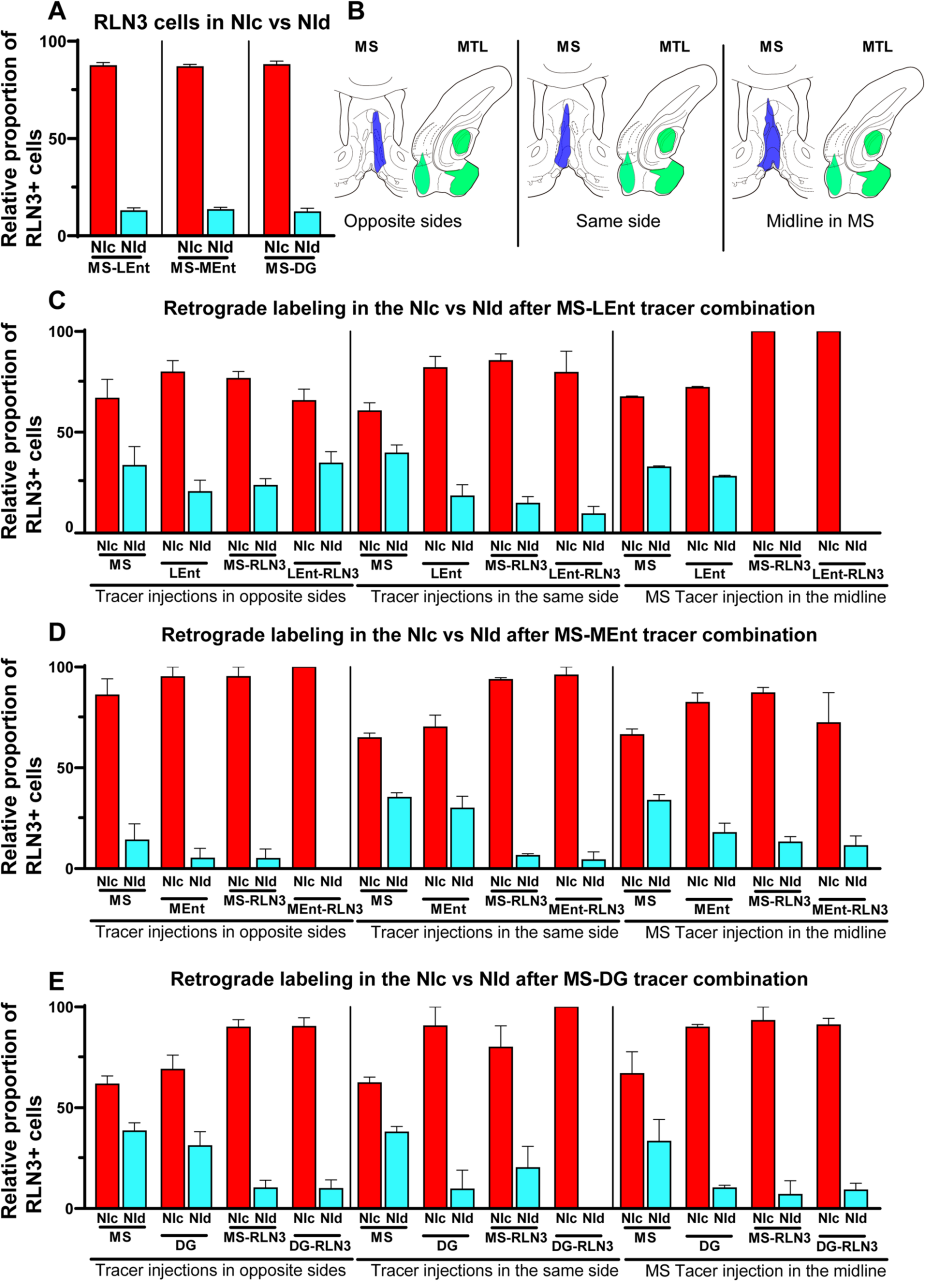
Comparative distribution of RLN3 in retrogradely labeled neurons in the NIc and NId after all combinations of injections. **A** Distribution of RLN3-positive cells in the NIc and NId from the three groups of injections; as expected there were no differences between them. **B** Diagram illustrating the laterality of the three combinations of injections either in opposite sides or in the same side or the MS injection in the midline. **C** Comparative distribution of labeling in the NIc and NId after the combination of tracer injections in the MS and the LEnt. **D** Comparative distribution of labeling in the NIc and NId after the combination of tracer injections in the MS and the MEnt. **E** Comparative distribution of labeling in the NIc and NId from the combination of tracer injections in the MS and the DG

Assessment of the occurrence of RLN3 immunolabeling in the two NI subdivisions revealed that 87.3 ± 0.97% (mean ± SEM) of RLN3 + neurons were located in the NI pars compacta (NIc), while 12.7 ± 0.97% were located in the NI pars dissipata (NId), and these proportions were unaffected by the experimental manipulations (Fig. 7A).

In order to assess the contribution of RLN3 + vs RLN3– neurons in the NIc or NId to the projection to the MS or MTL, a comparison was conducted of labeling with a single tracer and two tracer/RLN3 labeling following tracer injections into the same or opposite sides of the brain, or into the midline MS (affecting both sides; Fig. 7B). Single tracer labeling in the NI from injections into the MS resulted in a smaller difference in labeling between NIc and NId than RLN3 labeling in these regions (compare Fig. 6A with MS columns in Fig. 7C–E). As not all sample series displayed normality, we employed Mann–Whitney (M–W), non-parametric median comparisons. The difference between the number of RLN3 + neurons in the NIc and NId was larger than the difference between retrograde labeling resulting from MS injections between the NIc and NId under the three conditions (i.e. combinations of tracer injections in the MS and LEnt (*p* = 0.0001, M–W *U* = 10), injections in MS and MEnt (*p* = 0.0004, M–W *U* = 10) and injections in MS and DG (*p* < 0.0001, M–W *U* = 10). By contrast, the number of neurons double-labeled with tracer and RLN3 displayed no significant difference between NIc and NId, compared to the RLN3 distribution in the NIc and NId (compare Fig. 6A with MS-RLN3 columns in Fig. 6C–E). These findings indicate that most NId neurons were RLN3 positive, although they were present in a lower density than NIc neurons.

No significant differences were observed in the contribution of the NIc or NId between RLN3 + and RLN3– neurons to the projections to the LEnt (Fig. 7C), MEnt (Fig. 7D) and DG (Fig. 7E; Table 2). These proportions were independent of whether labeling was the result of tracer injections into the same or opposite sides of the brain or into the midline MS (affecting both sides).

#### Laterality of ascending NI projections

We also compared the laterality of these projections. This process was quite complex, as some of the injections into the MS were into the contralateral side, while some were into the midline and affected both sides of the region (Suppl. Fig. 1). However, the fact that midline MS injections resulted in more or less equivalent labeling in the ipsilateral and contralateral NI, irrespective of the tracer used, supports the reliability of the data (Suppl. Fig. 1A-C). In general, the ascending projections from the NI to the MS and medial temporal lobe structures were highly lateralized. Some 60–80% of the projections of labeled neurons appeared on the ipsilateral side, while 20–40% appeared in the contralateral NI, and when the injections were into opposite sides, the retrograde-labeling also appeared in the opposite NI side, in similar proportions (Suppl. Fig. 1). No differences were observed between the MS-Lent, MS-MEnt or MS-DG combinations. Thus roughly 70% of retrogradely labeled neurons appeared in the ipsilateral side independent of whether the tracer was injected into the MS, MEnt or LEnt and no differences were observed between RLN3 + and RLN3– neurons.

### Electrophysiology experiments

**Fig. 8:**
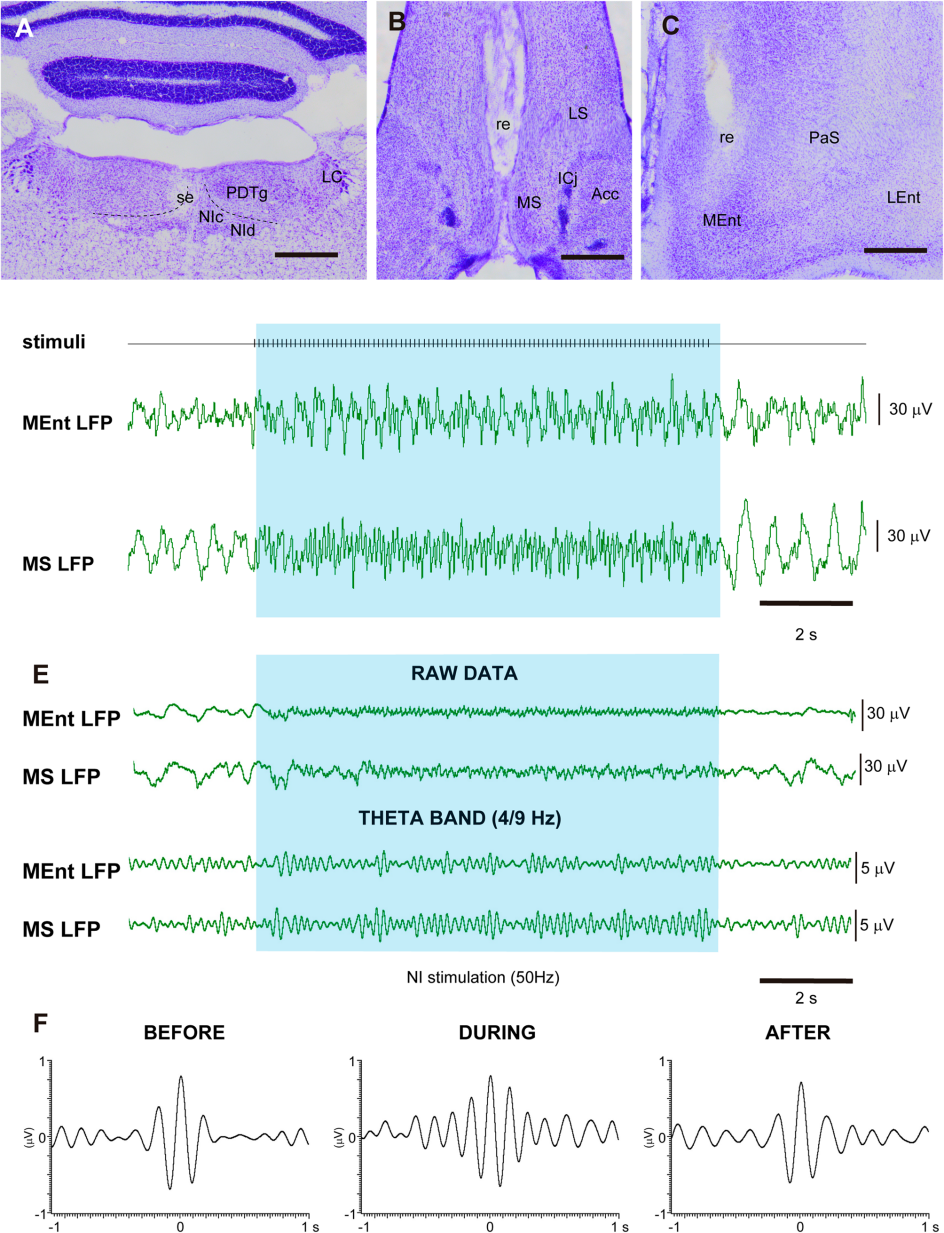
Effect of NI stimulation on MS and MEnt LFP. **A** Location of the stimulating electrode in the NI. **B** Location of the recording electrode in the MS. **C** Location of the recording electrode in the MEnt. **D** Representative LFP trace before, during and after NI stimulation. **E** Another representative LFP trace as raw data and after filtering to 4–9 Hz corresponding to theta activity. **F** Cross correlation of MS and MEnt activity before during and after NI stimulation

In these studies, we used a combination of electrical stimulation of the NI and field potential recordings in the MS and MEnt (Fig. 8A–C) before, during and after NI stimulation. The intensity of NI stimulation was set at twice the threshold to elicit theta rhythm in the hippocampal field potential for analysis (50–200 µA). During the period of NI stimulation (blue background) slow waves were reduced and theta waves were more prominent (Fig. 8D–E). The increase of theta activity was clearer in the filtered (4–9 Hz) recordings (Fig. 8E, lower records). The cross-correlograms of the theta rhythms recorded in MS and MEnt cortex indicated that both rhythms were in phase in all conditions (Fig. 8F). In all cases the peak of the cross-correlation was displaced 5–10 ms on the right indicating a small delay in the MEnt cortex with respect to the rhythm recorded in MS.

**Fig. 9:**
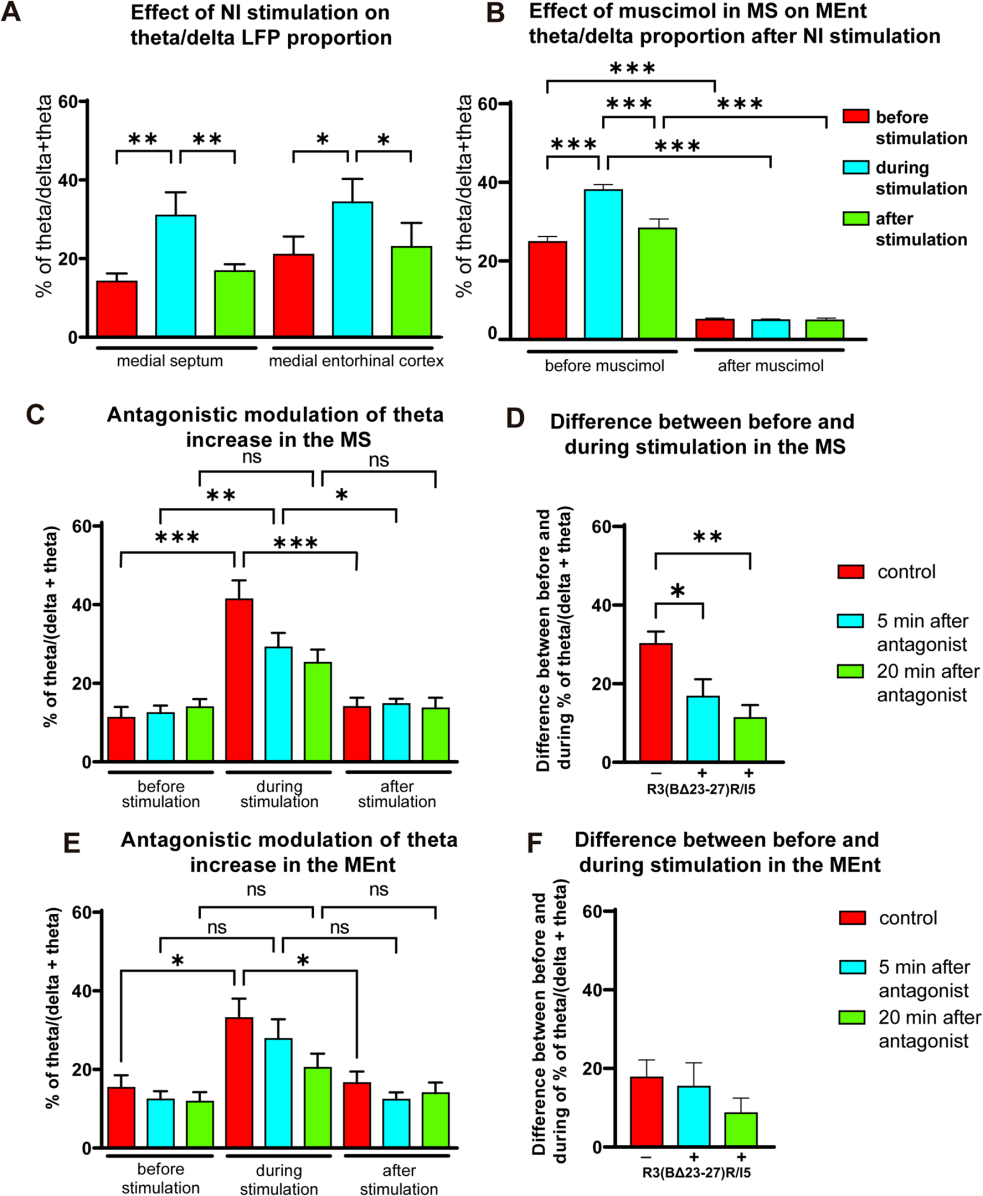
Effect of NI stimulation on MS and MEnt theta activity and disruption of theta by MS manipulation. **A** Effect of NI stimulation on the proportion of theta in the MS and MEnt. NI stimulation increased theta rhythm in both structures. **B** Effect on MEnt activity of muscimol inactivation of the MS. NI stimulation was unable to induce theta rhythm in the MEnt after MS inactivation with muscimol. **C**, **D** Effect of injection of the RXFP3 antagonist, R3(BΔ23-27)R/I5 (0.1 μl; 1 μg/μl), into the MS on the increase in the theta/(theta + delta) proportion in (**A**, **B**) MS, and (**C**, **D**) MEnt (*n* = 9 rats). **C** Effect of the RXFP3 antagonist on the increase and decrease in the theta proportion in the MS before, during and after NI stimulation, under the conditions of no antagonist (control), and 5 and 20 min after antagonist administration. **D** Histogram displaying the differences between before and during the NI stimulation under different conditions. **E** Effect of the RXFP3 antagonist on the increase/decrease of the theta proportion in the MEnt before, during and after NI stimulation, under the conditions of no antagonist, and 5 and 20 min after the MS administration of RXFP3 antagonist. **F** Histogram displaying the differences between before and during the NI stimulation under these conditions

Electrical stimulation of the NI induced theta rhythm in the MS and the MEnt (6.4 ± 1.2 or 5.8 ± 1.9 Hz, respectively). Data displayed normality in a Shapiro–Wilk test. In the MS, the percentage of the theta band (4–9 Hz) in the power spectrum (see “Methods”) was 14.6 ± 2.2% and this increased significantly to 32.7 ± 5.9% during the application of the NI stimulation train (*p* = 0.03, *t*_10_ = 3.952; paired *t* test). Theta power returned to control values 10 s after the stimulation train (17.7 ± 1.8%; *p* = 0.008, *t*_10_ = 3.511; paired *t* test; Fig. 9A). Similarly, the percentage of the theta band (4–9 Hz) of the power spectrum in the MEnt was 22.9 ± 4.6% and this increased significantly to 37.0 ± 5.9% (*p* = 0.016, *t*_10_ = 2.888; paired *t* test) during application of the NI stimulation train and returned to control values 10 s after the stimulation train (25.6 ± 6.2%; *p* = 0.008, *t*_10_ = 2.345; Fig. 9A).

The increase in theta rhythm evoked by NI stimulation was abolished when muscimol was injected into the MS (0.1 μl; 5 mM; Fig. 9B). Under control conditions, the percentage of the theta band in the MEnt was 24.8 ± 1.4% and this increased to 38.1 ± 1.4% during the application of the NI stimulation train (p = 0.0028, *n* = 8), while after muscimol, the theta band was reduced to 5.1 ± 0.4% before the stimulation train and was similar (4.9 ± 0.3%) during the NI stimulation train (Fig. 9B). Thus, the theta rhythm evoked in the MEnt by electrical stimulation of the NI was associated with an effect within the MS and not with a direct effect of NI on the MEnt.

In studies to establish whether the effect of NI stimulation was due, in full or in part, to activation of RXFP3 in MS, the RXFP3 antagonist, R3(BΔ23-27)R/I5 (0.1 μl; 1 μg/μl) was injected through a cannula targeting the MS. The RXFP3 antagonist strongly reduced the NI-evoked increase in the proportion of theta rhythm recorded in the MS and MEnt (Fig. 9C–F).

In the MS, under control conditions, NI stimulation increased the theta proportion in the LFP and this recovered to control values after the stimulation. This pattern of an increase and decrease in the proportion of theta rhythm after NI stimulation was altered after MS administration of R3(BΔ23-27)R/I5 (0.1 μl; 1 μg/μl; F(3.691, 29.53) = 17,13; *p* < 0.001, *n* = 9). Five min after the application of the antagonist a significant increase–decrease of the theta proportion was observed (Bonferroni test; *p* < 0.001 and *p* = 0.04, for control and 5 min, respectively). However, this increase–decrease in the theta proportion was not significant when the RXFP3 antagonist was applied 20 min before the NI stimulation and LFP recordings (Fig. 9A), consistent with a time-dependent effect of RXFP3 inhibition in the MS on theta rhythm. Thus, the application of the antagonist (R3(BΔ23-27)R/I5, 200 ng, ~ 40 pmol) reduced the increase in theta from 30.1 ± 3.2 to 16.7 ± 4.4% 5 min after the peptide injection and significantly to 11.3 ± 3.3% 20 min after the peptide treatment (F(1.688, 13.50) = 12,54; *p* = 0.0012, *n* = 9; one-way ANOVA; and post-hoc Bonferroni test for control and 5 min (*p* = 0.03) and control and 20 min (*p* = 0.003; Fig. 9D).

The effect was quite marked in the MEnt (one-way ANOVA; *p* < 0.001; F(4.288, 36.07) = 10.29, *n* = 9). There was a significant difference between before and during NI stimulation (*p* = 0.04) and during and after stimulation (*p* = 0.05), but no significant changes were observed in the MEnt theta proportion after the MS injection of the RXFP3 antagonist either 5 min or 20 min after the injection (Fig. 9E). A comparison of the differences between before and during NI stimulation under the three conditions revealed non-significant changes (small decreases; Fig. 9F).

## Discussion

**Fig. 10:**
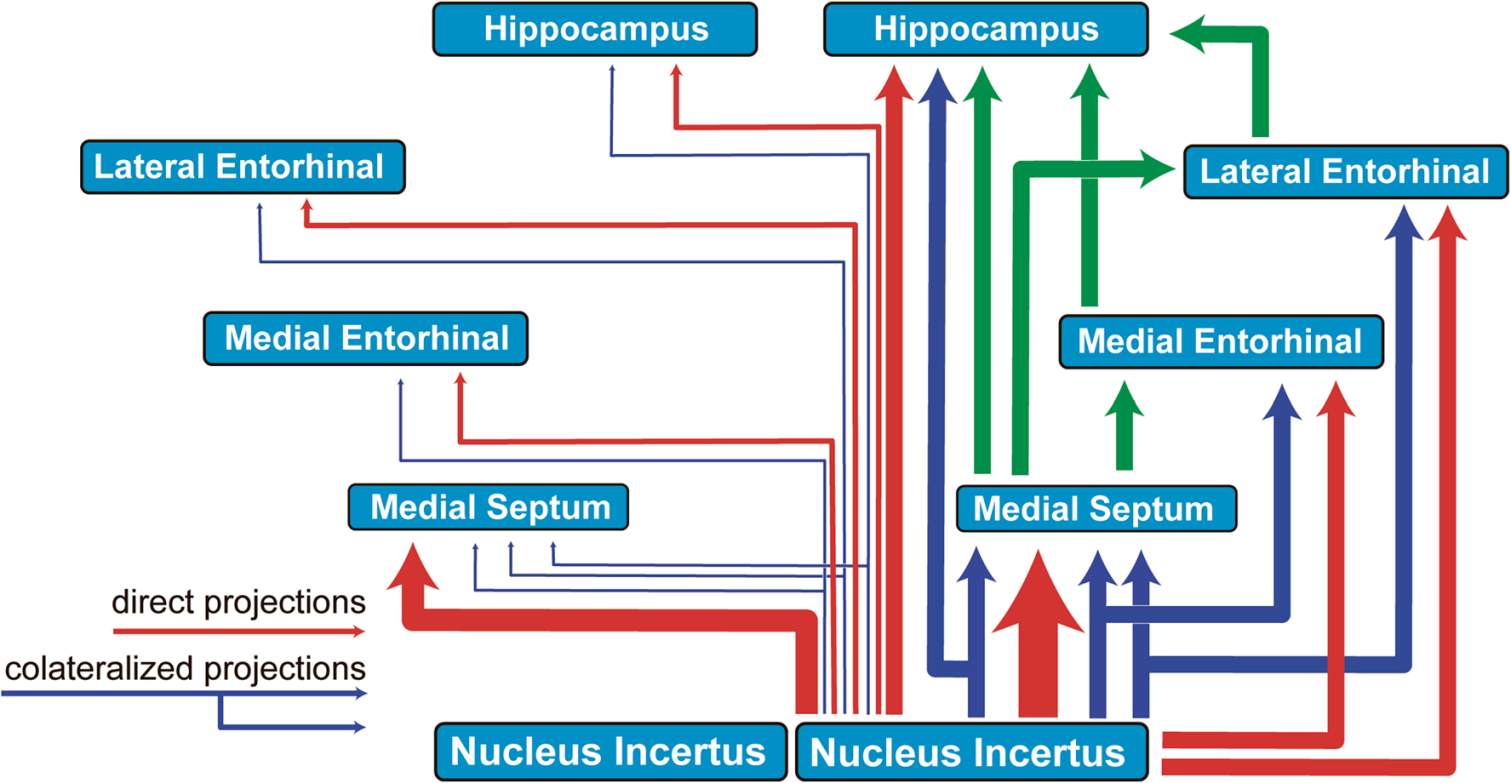
A model based on the pattern of direct, collateralized and bilateral projections from the NI to the MS and medial temporal lobe, including the MEnt, LEnt and DG as part of the hippocampal formation. This model illustrates that the MS is the main relay station from the NI to the hippocampus, but the ascending connections of these projections are rather heterogeneous, and overall, have a high level of collateralization. Red arrows represent direct projections and blue arrows collateralized projections from the ipsilateral NI; and green arrows represent consecutive ascending projections from the targeted areas to the hippocampus

The current studies provide anatomical and physiological evidence that the MS is the central neuronal hub that receives the majority of ascending NI projections to the hippocampus and the MEnt in the rat (Fig. 10). From an anatomical viewpoint, ascending projections from the NI have a very low level of collateralization, and segregated populations of neurons project to MS, LEnt, MEnt and DG. Additionally, the RLN3 + subpopulation of NI neurons collateralize more (~ 5% in RLN3– and ~ 14% in RLN3 + neurons) and are less likely to project to the MS than to the MTL (proportion of ~ 70/30% of projections to the MS vs MTL in RLN3– neurons and ~ 60/40% of projections to MS vs MTL in RLN3 + neurons). Similarly, we have shown a strong effect of NI electrical stimulation on theta rhythm in the MS, and observed that the inhibition of RLN3/RXFP3 signaling in the MS abolished the theta activity evoked in the MEnt by NI stimulation. These data reflect the critical involvement of the MS as a hub that conveys the influence of NI projections to the hippocampus and entorhinal cortex, and the ability of RXFP3 activation in the area to drive neural synchronization in the theta band.

Several studies have described heterogeneity in the chemical markers expressed by NI neurons in the rat, which include RLN3, CCK, neuromedin-B, GAD, calretinin (CR) and calbindin-28kD (CB) (Ryan et al. 2011; Ma et al. 2017b). Previous observations indicate that most, but not all, GAD-positive (GABAergic) neurons are RLN3 + and conversely, that most, but not all, RLN3 neurons were GAD-positive; while only 30% of NI neurons are CB-positive (Ma et al. 2007). In subsequent studies it was reported that ~ 40% of RLN3 neurons were positive for both CB and CR, ~ 15% were positive for CB and RLN3, ~ 25% were positive for CR and RLN3 and ~ 19% of RLN3 neurons did not co-express CB or CR (Szlaga et al. 2022). In contrast, RLN3 and CCK neurons in the rat NI are segregated populations of neurons (Szlaga et al. 2022); while in the mouse NI RLN3 neurons are a subpopulation of neuromedin-B neurons (Lu et al. 2020). Our study supports this heterogeneity in the NI based on the patterns of ascending anatomical connections. There are currently no data regarding the correspondence between the chemical and connective heterogeneity, but our data indicate that the RLN3 neurons are different from the other NI neuronal types.

Furthermore, in light of the expression of the corticotropin-releasing factor receptor 1 (CRFR1) by NI neurons (Bittencourt and Sawchenko 2000; Van Pett et al. 2000; Tanaka et al. 2005; Banerjee et al. 2010), the NI is implicated in stress responses. In an earlier study on the heterogeneity of NI neurons in the rat, CRFR1-like immunoreactivity was present in ~ 50% of NI neurons and 50% of these neurons expressed RLN3 (Ma et al. 2013). Based on this expression profile, RLN3 neurons account for ~ 25% of all NI neurons. In the current studies, RLN3 neurons accounted for ~ 25% of the neurons projecting to MS, MEnt, LEnt or DG. This heterogeneity of NI neurons was further identified by studying the effect of CRF application on NI neuronal activity (Ma et al. 2013). Using in vivo recordings, it was observed that most RLN3 + neurons responded to intracerebroventricular (icv) CRF infusion by increasing their firing rate. By contrast, most RLN3– neurons were inhibited by icv CRF infusion. Thus, specific RLN3 neurons may activate ascending projections to the telencephalic targets in response to CRF under stressful conditions.

Regarding the nature of the classical neurotransmitter in NI and NI RLN3 neurons, it was originally thought that GAD-positive (GABAergic) neurons were concentrated in the NI, unlike the posterodorsal tegmentum (Olucha-Bordonau et al. 2003). Thereafter, most, but not all, RLN3 neurons were reported to express GAD (i.e. GABAergic) and project to MS (Ma et al. 2007). However, it was also demonstrated that vGlut2-positive neurons in the NI project to the MS (Cervera-Ferri et al. 2012). The possibility of glutamatergic transmission in the projection from the NI to the MS has also been shown recently in an experiment combining retrograde viral tracing with in situ fluorescent hybridization detection of vGlut2 mRNA in rats. MS injection of retrograde AAV-mCherry resulted in 38% co-localization of mCherry in NI neurons expressing vGlut2 mRNA that were preferentially located in the NId. In this study, 20% of the neurons projecting to the MS expressed RLN3 mRNA, which was a similar proportion to that observed in the current study. Finally, of the RLN3 neurons projecting to the MS, 60% contained vGAT mRNA and 40% expressed vGlut2 mRNA (Szlaga et al. 2022).

Additionally, it has been demonstrated that NI activation simultaneously induces arousal, locomotion, and hippocampal theta rhythm. NI microstimulation induced increased velocity, mobility and rotations in freely moving rats (Farooq et al. 2016). Chemogenetic (CNO-DREADD) activation of the NI induced locomotion as well as increased cortical theta activity and vigilance behavior in rats (Ma et al. 2017a). Furthermore, optogenetic activation of neuromedin-B NI neurons in mice resulted in increased locomotion, arousal and hippocampal theta rhythm (Lu et al. 2020).

There are currently little data on the differential neurochemical features of neurons in the two NI subnuclei, NIc and NId, other than the restricted localization of CCK neurons in the NIc (Olucha-Bordonau et al. 2003). However, structural differences are observed between NIc and NId in rats and mice, as subnuclei are clearly identified in rats, while limits are more difficult to identify in mice. Additionally some peptides like neuromedin-B appear to be more concentrated in neurons within the lateral than the medial region in mice (Lu et al. 2020). We have observed that RLN3 + neurons in the NIc and NId are ~ 75% and ~ 25% of the total populations, respectively; but we have noted that this difference is significantly lower among double-labeled neurons projecting to either MS or MTL. Thus, NId neurons appear to be more likely to project to telencephalic targets, such as MS or MTL.

Our findings indicate that the theta rhythm evoked in the entorhinal cortex by NI stimulation was due to activation of MS neurons. The fact that the peak of the correlogram is displaced by 5–10 ms supports the finding that there is a delay between the rhythms in MS and Ent. Previous findings have shown that layer II of the entorhinal cortex is a generator of theta and gamma activities (Dickson et al. 2000). However, when theta rhythm is generated from the NI, the main site of generation is in the MS, even though there are direct projections from NI to the cortex. Early electrophysiological studies identified the occurrence of two types of NI neurons displaying spontaneous activity under urethane anesthesia, i.e. type I, theta-tonic neurons and type II, fast-firing beta rhythmic neurons (Nuñez et al. 2006). In a subsequent study, a type III neuron was observed, which was silent under baseline conditions and increased its spike rate regularity and phase-locking to stimuli that induce hippocampal theta (Martínez-Bellver et al. 2015). Similarly, in patch-clamp studies, at least two types of NI neurons have been observed, i.e. type I, which display a delay in return to baseline after the application of a hyperpolarizing current pulse, an effect that depends on an A-type potassium current (Blasiak et al. 2015); and type II neurons, which display a rebound depolarization after a hyperpolarizing pulse, which is dependent on a voltage-gated calcium current (Blasiak et al. 2015). Most RLN3 neurons are type I and thus express A-type potassium channels, while notably, none of the type II neurons have been identified as RLN3 + (Blasiak et al. 2015). Type I NI neurons are also characterized by a lower resting potential, longer interspike intervals and more regular firing than type II neurons, and the MS is predominantly innervated by type I neurons (Szlaga et al. 2022).

The MS has previously been proposed as a major relay hub for NI projections to the hippocampus, and MS infusion of an RXFP3 agonist directly induced hippocampal theta (Ma et al. 2009a). The MS neurons targeted by NI projections, including RLN3 + projections, have been examined in detail (Olucha-Bordonau et al. 2012), and several MS neuronal types are targeted by RLN3-containing fibers that mainly form symmetric contacts on MS neuronal bodies and processes. In addition, the connections are reciprocal, and calretinin-positive neurons in the MS project to the NI (Sánchez-Pérez et al. 2015). It has also been observed that GABAergic, but not cholinergic neurons in the rat MS express RXFP3 mRNA (Albert-Gascó et al. 2017), while both glutamatergic and GABA-ergic MS neurons express RXFP3 mRNA in mice (Haidar et al. 2019). In terms of signaling effects, the intracerebroventricular (icv) infusion of the RXFP3 agonist, RXFP3-A2, indirectly activated the Erk1/2 pathway in cholinergic neurons of the MS, which lack RXFP3 mRNA (Albert-Gascó et al. 2017). In this sense, it has been found that local glutamatergic circuits may activate both cholinergic (Hajszan et al. 2004) and parvalbumin-positive, GABAergic (Wu et al. 2004) neurons; and GABAergic interneurons contribute to intrinsic connectivity underlying hippocampal theta rhythmogenesis through septohippocampal pathways (Leão et al. 2015). Another previous anatomical study also demonstrated that axons/terminals from NI/RLN3 neurons make close contact with septal GABAergic neurons, including those that project to the hippocampus (Ma et al. 2009a, b). Therefore, these terminals in the MS likely act on the different types of neurons that contribute to hippocampal theta rhythm generation (see Nuñez and Buño 2021).

The behavioral effect of disrupting RLN3/RXFP3 transmission in the MS was originally tested pharmacologically. MS infusion of the RXFP3 antagonist, R3(BΔ23-27)R/I5, impaired performance in a spatial spontaneous alternation task (Ma et al. 2009a), a form of spatial working memory. The effect of the ascending RLN3 projections to the MS and DG can be also be inferred by results in transgenic mice expressing a floxed RXFP3 (Haidar et al. 2017, 2019). Deletion of the RXFP3 sequence through AAV-Cre injection into the MS, which removed RXFP3 from both glutamatergic and GABAergic neurons, resulted in impairment of both acquisition and long-term reference memory in a Morris water maze (MWM) (Haidar et al. 2019). By contrast, in this same mouse line, deletion of RXFP3 expression in the hilar region of the dentate gyrus did not result in impairment of spatial memory performance in the MWM, but affected alternation in the T-maze test (Haidar et al. 2017).

Together these data indicate that the NI is a heterogeneous collection of neurons displaying different neurochemical, neurophysiological, and anatomical properties. Our findings indicate that their level of collateralization to specific target regions in the MS and MTL is quite low, which indicates that NI neurons mainly project to specific, single target regions. Furthermore, specific groups of NI cells such as RLN3/GABAergic neurons display a different and specific level of collateralization. The most prominent target of the NI by weight of projections is the MS and via this region, the NI contributes to driving and modulation of hippocampal and entorhinal theta rhythm. Notably, pharmacological blockade of RXFP3 in the MS strongly disrupts theta rhythm in the hippocampus.

## Supplementary Information

Below is the link to the electronic supplementary material.

Supplementary file1 (TIF 2973 KB) Comparative distribution of traced neurons in the ipsilateral and contralateral sides after tracer injections into the MS or the MTL. A. Occurrence of ipsilateral (I) or contralateral (C) NI labeling after combined injections in the MS and LEnt. Groups with injections in opposite sides, in the same side or in the midline MS affecting both sides were analyzed. B. Laterality of the retrograde-labeling in the NI after a combination of injections in the MS and the MTL. C. Laterality of the retrograde-labeling in the NI after a combination of injections in the MS and DG.

Supplementary file2 (DOCX 17 KB)

## Data Availability

The datasets generated during or analyzed during the current study are available in the handle repository of the UJI http://hdl.handle.net/10234/201305.

## Abbreviations

4V: 4th ventricle
Acc: Nucleus accumbens
CTB: Cholera toxin B subunit
DG: Dentate gyrus
F: Female
FG: Fluorogold
iCj: Nucleus of the insula of Calleja
LEnt: Lateral entorhinal cortex
LFP: Local field potential
LS: Lateral septum
M: Male
MEnt: Medial entorhinal cortex
mlf: Medial longitudinal fasciculus
MPO: Medial preoptic area
MS: Medial septum
MTL: Medial temporal lobe
Nic: Nucleus incertus pars compacta
NId: Nucleus incertus pars dissipata
PDTg: Posterodorsal tegmental nucleus
PRh: Perirhinal cortex
RLN3: Relaxin 3
Rxfp3: Relaxinfamily protein receptor 3
ST: Bed nucleus of the stria terminalis
