# Supplementary figures and images for: Nucleus incertus projections to rat medial septum and entorhinal cortex: rare collateralization and septal-gating of temporal lobe theta rhythm activity
Source: Brain Struct Funct. 2023 May 12;228(5):1307–28. doi: 10.1007/s00429-023-02650-x (PMC10250478; doi:10.1007/s00429-023-02650-x)

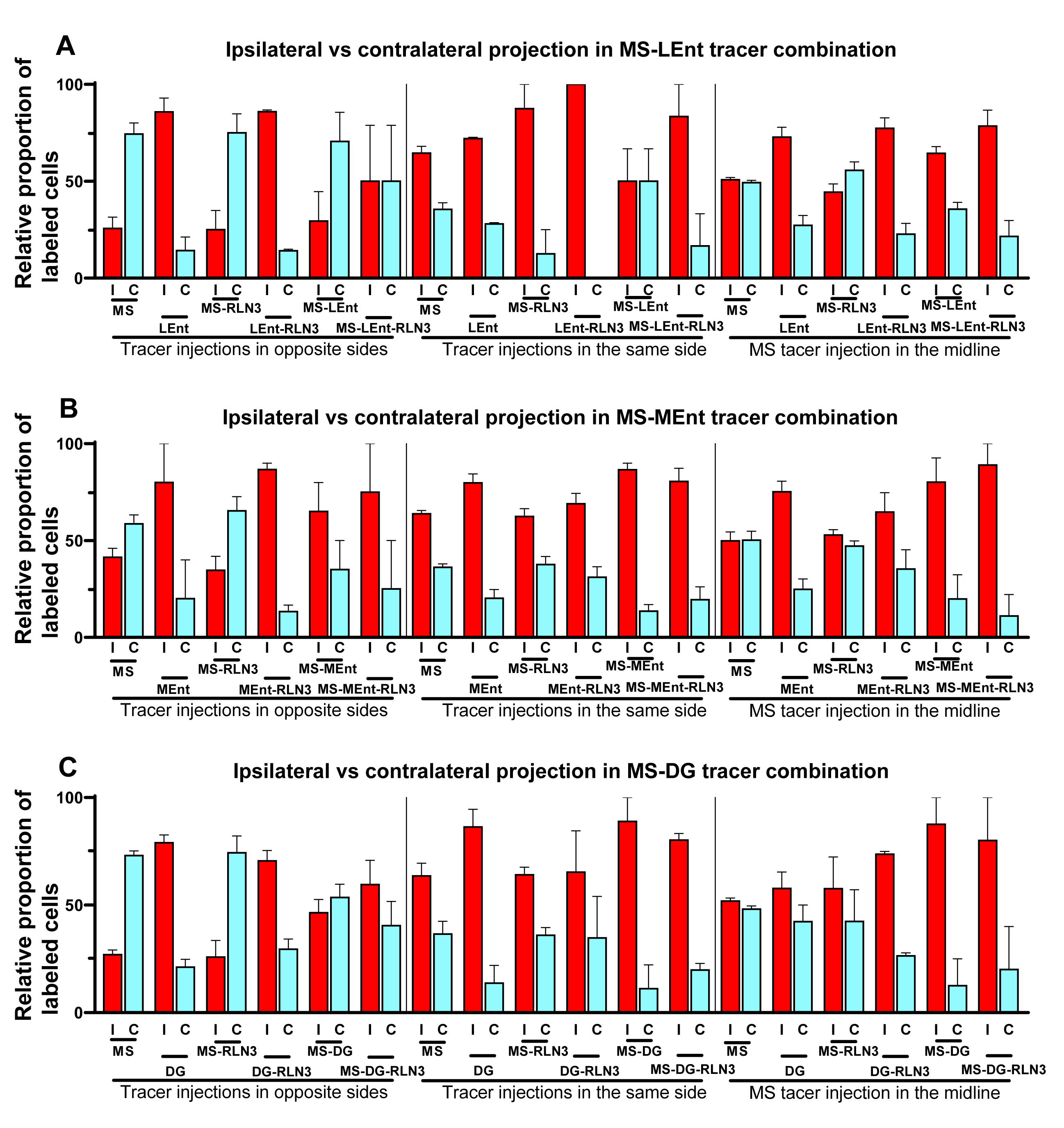

Supplement: Supplementary file 1 — Supplementary file1 (TIF 2973 KB) Comparative distribution of traced neurons in the ipsilateral and contralateral sides after tracer injections into the MS or the MTL. A. Occurrence of ipsilateral (I) or contralateral (C) NI labeling after combined injections in the MS and LEnt. Groups with injections in opposite sides, in the same side or in the midline MS affecting both sides were analyzed. B. Laterality of the retrograde-labeling in the NI after a combination of injections in the MS and the MTL. C. Laterality of the retrograde-labeling in the NI after a combination of injections in the MS and DG. [file 429_2023_2650_MOESM1_ESM.tif]
