# Supplementary material for: Nucleus incertus projections to rat medial septum and entorhinal cortex: rare collateralization and septal-gating of temporal lobe theta rhythm activity
Source: Brain Struct Funct. 2023 May 12;228(5):1307–28. doi: 10.1007/s00429-023-02650-x (PMC10250478; doi:10.1007/s00429-023-02650-x)
Supplement: Supplementary file 2 — Supplementary file2 (DOCX 17 KB) [file 429_2023_2650_MOESM2_ESM.docx]

**Supplementary Table 1**. Relative percentages of retrogradely-traced neurons that were or were not positive for RLN3 and their differences in the three combinations of MS/LEnt, MS/MEnt and MS/DG. Percentages are entered as mean ± standard error of the mean.

| **MS/LEnt Combinations** | | | |
| --- | --- | --- | --- |
| MS | | LEnt | |
| MS/RLN3– | MS/RLN3+ | LEnt/RLN3– | LEnt/RLN3+ |
| 76.4 ± 1.9 | 23.6 ± 1.9 | 60.7 ± 4.3 | 39.3 ± 4.3 |
| **MS/MEnt Combinations** | | | |
| MS | | MEnt | |
| MS/RLN3– | MS/RLN3+ | MEnt/RLN3– | MEnt/RLN3+ |
| 79.6 ± 2.5 | 20.4 ± 2.5 | 71.0 ± 3.5 | 28.9 ± 3.5 |
| **MS/DG Combinations** | | | |
| MS | | DG | |
| MS/RLN3– | MS/RLN3+ | DG/RLN3– | DG/RLN3+ |
| 79.2 ± 2.9 | 20.8 ± 2.9 | 67.8 ± 4.0 | 35.3 ± 4.0 |
